# Insight into the practical performance of RT-PCR testing for SARS-CoV-2 using serological data: a cohort study

**DOI:** 10.1016/S2666-5247(20)30200-7

**Published:** 2021-02

**Authors:** Zhen Zhang, Qifang Bi, Shisong Fang, Lan Wei, Xin Wang, Jianfan He, Yongsheng Wu, Xiaojian Liu, Wei Gao, Renli Zhang, Wenfeng Gong, Qiru Su, Andrew S Azman, Justin Lessler, Xuan Zou

**Affiliations:** a Department of Public Health Information, Shenzhen Center for Disease Control and Prevention, Shenzhen, China; b Department of Pathogenic Biology, Shenzhen Center for Disease Control and Prevention, Shenzhen, China; c Department of Communicable Diseases Control and Prevention, Shenzhen Center for Disease Control and Prevention, Shenzhen, China; d Department of Epidemiology, Johns Hopkins Bloomberg School of Public Health, Baltimore, MD, USA; e Shenzhen Center for Disease Control and Prevention, Shenzhen, China; f The Bill & Melinda Gates Foundation, Seattle, WA, USA; g Pediatric Research Institute, Shenzhen Children's Hospital, Shenzhen, China

## Abstract

**Background:**

Virological detection of severe acute respiratory syndrome coronavirus 2 (SARS-CoV-2) through RT-PCR has limitations for surveillance. Serological tests can be an important complementary approach. We aimed to assess the practical performance of RT-PCR-based surveillance protocols and determine the extent of undetected SARS-CoV-2 infection in Shenzhen, China.

**Methods:**

We did a cohort study in Shenzhen, China and attempted to recruit by telephone all RT-PCR-negative close contacts (defined as those who lived in the same residence as, or shared a meal, travelled, or socially interacted with, an index case within 2 days before symptom onset) of all RT-PCR-confirmed cases of SARS-CoV-2 detected since January, 2020, via contact tracing. We measured anti-SARS-CoV-2 antibodies in serum samples from RT-PCR-negative close contacts 2–15 weeks after initial virological testing by RT-PCR, using total antibody, IgG, and IgM ELISAs. In addition, we did a serosurvey of volunteers from neighbourhoods with no reported cases, and from neighbourhoods with reported cases. We assessed rates of infection undetected by RT-PCR, performance of RT-PCR over the course of infection, and characteristics of individuals who were seropositive on total antibody ELISA but RT-PCR negative.

**Findings:**

Between April 12 and May 4, 2020, we enrolled and collected serological samples from 2345 (53·0%) of 4422 RT-PCR-negative close contacts of cases of RT-PCR-confirmed SARS-CoV-2. 1175 (50·1%) of 2345 were close contacts of cases diagnosed in Shenzhen with contact tracing details, and of these, 880 (74·9%) had serum samples collected more than 2 weeks after exposure to an index case and were included in our analysis. 40 (4·5%) of 880 RT-PCR-negative close contacts were positive on total antibody ELISA. The seropositivity rate with total antibody ELISA among RT-PCR-negative close contacts, adjusted for assay performance, was 4·1% (95% CI 2·9–5·7), which was significantly higher than among individuals residing in neighbourhoods with no reported cases (0·0% [95% CI 0·0–1·1]). RT-PCR-positive individuals were 8·0 times (95% CI 5·3–12·7) more likely to report symptoms than those who were RT-PCR-negative but seropositive, but both groups had a similar distribution of sex, age, contact frequency, and mode of contact. RT-PCR did not detect 48 (36% [95% CI 28–44]) of 134 infected close contacts, and false-negative rates appeared to be associated with stage of infection.

**Interpretation:**

Even rigorous RT-PCR testing protocols might miss a substantial proportion of SARS-CoV-2 infections, perhaps in part due to difficulties in determining the timing of testing in asymptomatic individuals for optimal sensitivity. RT-PCR-based surveillance and control protocols that include rapid contact tracing, universal RT-PCR testing, and mandatory 2-week quarantine were, nevertheless, able to contain community spread in Shenzhen, China.

**Funding:**

The Bill & Melinda Gates Foundation, Special Foundation of Science and Technology Innovation Strategy of Guangdong Province, and Key Project of Shenzhen Science and Technology Innovation Commission.

## Introduction

Virological detection of severe acute respiratory syndrome coronavirus 2 (SARS-CoV-2) through RT-PCR is the gold standard for diagnosing infection.^1^ Almost all diagnostic testing for COVID-19 is done using PCR-based methods. Like all virological tests, RT-PCR has imperfect sensitivity,^2^, ^3^, ^4^ and patterns of viral shedding mean that the chance of testing positive varies over the course of infection.^5^, ^6^ Hence, although RT-PCR might be highly accurate at identifying those who are currently infectious, individuals must be tested at the right time during their infection to be detected, which reduces the utility of virological testing for measuring overall SARS-CoV-2 incidence. Serological tests offer an alternative approach for detecting SARS-CoV-2 infection by measuring circulating antibodies against the virus. By contrast with virological tests, serological tests can detect if an individual has been infected even months after viral clearance, though serological tests also have imperfect sensitivity and specificity.^7^

By utilising both tests in the same population, we can gain an understanding of the practical performance of RT-PCR-based surveillance, if three conditions are met. First, virological RT-PCR surveillance must have occurred around the time of potential exposure in the population. Second, the same individuals tested by RT-PCR must later receive serological tests. Third, there must be a low chance of infection between the periods of virological and serological surveillance.

**Research in context**

**Evidence before this study**

We searched PubMed on Sept 3, 2020, with no date or language restrictions, using the keywords “serology” AND “PCR” AND “SARS-CoV-2”, and found two articles that reported serological testing in a cohort of individuals who had been tested with RT-PCR. However, these studies either did not report results of serological testing among a group only consisting of RT-PCR-negative individuals, or did not provide sufficient information on the PCR-based surveillance protocol used to be able to assess the practical performance of PCR-based surveillance.

**Added value of this study**

This is the largest study, to our knowledge, that reports serological testing in a large group of RT-PCR-negative close contacts of cases of severe acute respiratory syndrome coronavirus 2 (SARS-CoV-2), in a setting where community transmission after initial RT-PCR testing was highly unlikely. We provide important new data on the practical performance of RT-PCR as a surveillance tool and show how new data add nuance to previous results on the variation in the performance of RT-PCR testing over the course of infection. We found that approximately 4% of PCR-negative close contacts of cases of SARS-CoV-2 were seropositive for anti-SARS-CoV-2 antibodies on ELISA. Therefore, despite a rigorous testing system in Shenzhen, China, virological RT-PCR testing did not detect approximately 30–40% of infections among close contacts of confirmed cases. We also found that seropositive contacts who had negative RT-PCR results were less likely to report symptoms than those who tested positive on RT-PCR, which reflects the difficulties in timing RT-PCR testing for optimal sensitivity.

**Implications of all the available evidence**

These results highlight the practical limitation of virological-based RT-PCR surveillance. Innovations to both improve the accuracy of virological testing and allow for more frequent, less invasive testing are greatly needed. However, we show that SARS-CoV-2 control is possible through rigorous and aggressive surveillance, isolation and quarantine, despite the limitations of virological testing.

For SARS-CoV-2, Shenzhen, China is one area where all three of these conditions were met. The Shenzhen Center for Disease Control and Prevention (CDC) implemented extensive contact tracing among local residents after the epidemic started in mid-January, 2020. Nearly all infections during the initial epidemic that receded around mid-February were among travellers and their close contacts.^8^ As of Aug 1, 2020, no local case had been imported since Feb 21, 2020.^9^ In April, we did serological testing among PCR-negative close contacts of confirmed cases of SARS-CoV-2 and did a serosurvey of local residents without known exposure to COVID-19. We aimed to compare the results of these serological analyses with those of the initial RT-PCR-based surveillance to gain insight into the practical performance of RT-PCR virological testing protocols, and to determine the extent of undetected infection in the region. Using RT-PCR results from infected individuals, we also aimed to characterise the false-negative rate of RT-PCR, both before and after symptom onset.

## Methods

### Study design and participants

Shenzhen is a city in southern China with a population of more than 12 million people. The Shenzhen CDC implemented a surveillance programme and strict quarantine policy to monitor travellers from Hubei province, China, where the first case of COVID-19 was detected, from early January, 2020, until the lockdown in Hubei province was lifted at around the end of March, 2020. Mandatory screening and quarantine of all international travellers started on March 27, 2020. Suspected cases were also detected at local hospitals and through fever screening in neighbourhoods. Contact tracing was used to identify close contacts of confirmed cases of SARS-CoV-2, defined as those who lived in the same residence as, or shared a meal, travelled, or socially interacted with, an index case within 2 days before symptom onset.^8^ Frequency of contact was defined as frequent if individuals interacted with those with an index case more than three times per week, moderate if two to three times per week, and rare if once per week. Nasopharyngeal swab samples were collected from individuals with suspected infection and their close contacts and were tested for SARS-CoV-2 by RT-PCR. By protocol, RT-PCR testing was required for all close contacts at the beginning of quarantine, and release was conditional on two consecutive negative RT-PCR tests from samples collected at least 1 day apart. Those not contacted within 14 days of the last day of putative exposure were tested once. Symptomatic individuals were isolated and treated at designated hospitals regardless of RT-PCR test results for a minimum of 14 days after the last day of putative exposure. All close contacts of confirmed cases, asymptomatic individuals who tested positive for SARS-CoV-2 on RT-PCR, and travellers from Hubei province (before lockdown was lifted at around the end of March, 2020) and abroad (after March 27, 2020) were quarantined at centralised facilities, and monitored for 14 days after the last day of putative exposure. After the lockdown in Hubei province was lifted, domestic travellers were required to test negative by RT-PCR before leaving Hubei province and upon arrival in Shenzhen.

Between April 12 and May 4, 2020, we attempted to recruit by telephone all RT-PCR-negative close contacts of all confirmed SARS-CoV-2 cases in Shenzhen for serological testing. RT-PCR test records before clinical diagnosis were available for all close contacts, as well as their time and mode of putative exposure. Some of these RT-PCR-negative close contacts were included in a previous study that characterised the epidemiology and transmission of COVID-19 in Shenzhen,^8^ hereafter referred to as the Shenzhen cohort (appendix p 4). In addition, between April 17 and April 23, 2020, we did a serosurvey of 350 volunteers from neighbourhoods where no cases were reported (in two districts, Luohu and Longgang), and 50 volunteers from neighbourhoods with reported cases (in Luohu; three RT-PCR-confirmed cases within case neighbourhoods; 1·2 cases per 10 000 population). The community serosurvey recruited the same number of volunteers in seven age groups (0–9, 10–19, 20–29, 30–39, 40–49, 50–59, and 60 years or older).

To estimate the sensitivity of RT-PCR tests over the course of infection, we obtained RT-PCR results and time of sampling for a subset of infected individuals (appendix p 4). Eligible individuals for this analysis had to have at least one positive RT-PCR or serological test, and have been detected through contact tracing, not through symptom-based surveillance. To be included in the estimates of sensitivity of RT-PCR based on time before or after symptom onset, individuals must also have reported symptoms during the initial investigation. For RT-PCR-confirmed cases, RT-PCR results were available up to the first positive, and potentially one additional confirmatory (from a different date), RT-PCR test.

All close contacts and neighbourhood residents in the serosurvey provided written informed consent before participating in the serological testing. Contact tracing and RT-PCR testing are part of the continuing public health investigation of the emerging outbreak of SARS-CoV-2 and therefore the individual informed consent was waived for these aspects of the study. The study was approved by the ethics committees of Shenzhen CDC. This study was done in support of an ongoing public health response, and hence was determined not to be human subjects research after consultation with the Johns Hopkins Bloomberg School of Public Health institutional review board.

### Procedures

Real-time RT-PCR assay was done using a SARS-CoV-2 nucleic acid detection kit (Shanghai BioGerm Medical Technology, Shanghai, China), which mainly targets the open reading frame 1ab (ORF1ab) and the nucleocapsid protein (N), according to the manufacturer's protocol. Results were considered positive if the cycle threshold value was less than 37 and the sample was positive for both ORF1ab and N genes, negative if the cycle threshold value was more than 40, and retesting was recommended otherwise. Nasopharnygeal swab samples were collected at local hospitals and clinics, as well as at ten district-level CDCs in Shenzhen. All positive samples had confirmatory testing by Shenzhen CDC.

We assessed anti-SARS-CoV-2 antibodies in participants' serum using commercially available total antibody, IgG, and IgM ELISAs (Beijing Wantai Biological Pharmacy Enterprise, Beijing, China) that detect antibodies binding to SARS-CoV-2 spike protein receptor binding domain, according to the manufacturer's protocol. Samples with an absorbance value of greater than the cutoff value were considered positive as per manufacturer recommendation. We excluded close contacts who were sampled within 2 weeks of last exposure (n=295), due to the low expected sensitivity of antibody tests during this time window.^10^ Previously published studies reported that the manufacturer's recommended cutoff for positivity of the total antibody ELISA had sensitivity ranging from 93% (validation cases sampled 7–21 or more days after symptom onset)^11^ to 99% (validation cases sampled 1–43 days after symptom onset),^12^ and specificity ranging from 99%^12^ to 100%^11^. A combined estimate of sensitivity from the two validation studies was calculated with inverse-variance weights.

We estimated the crude and adjusted seropositive rate for total antibody, IgG, and IgM ELISAs among RT-PCR-negative close contacts in the Shenzhen cohort, other RT-PCR-negative close contacts outside of the Shenzhen cohort, and local residents living in neighbourhoods with reported cases and in neighbourhoods with no reported cases. To account for assay performance, we calculated the adjusted seropositivity rate as (proportion of positive tests + [specificity – 1]) / (sensitivity + specificity – 1).^13^, ^14^, ^15^ We based our analyses on those seropositive on total antibody ELISA, unless otherwise specified.

### Statistical analysis

We used Poisson regression to estimate the relative risk of symptoms among seropositive close contacts compared with seronegative close contacts, and among RT-PCR-positive close contacts compared with RT-PCR-negative close contacts who were seropositive. We used the Breslow-Day test to assess heterogeneity in the exposure-specific odds ratio of positivity between serological and virological tests.^16^ The exposures included sex, age, contact frequency, and mode of contact. We estimated the number of infected close contacts not detected by RT-PCR using the adjusted seropositivity rate, estimated among all tested PCR-negative close contacts. Then, using the adjusted seropositivity rate among close contacts in Shenzhen, we re-estimated the secondary attack rate and effective reproductive number in the Shenzhen cohort using methods described previously.^8^ We assumed that the RT-PCR-negative close contacts with serological test results were representative of all RT-PCR-negative close contacts in the Shenzhen cohort.

We estimated sensitivity of RT-PCR over time from symptom onset using data from both RT-PCR-positive and seropositive symptomatic close contacts.^8^ To estimate sensitivity of RT-PCR over time from last exposure to an index case, we also included seroconverted contacts who were asymptomatic. Using an approach similar to that of Kucirka and colleagues,^5^ we fitted a Bayesian logistic regression model for test sensitivity with a polynomial spline for time since symptom onset. We assessed the performance of models that incorporated polynomial splines of third to fifth degree with widely applicable information criterion. We implemented this model in the Stan probabilistic programming language and used the *rstan* package to run the model and analyse outputs. We ran 6000 iterations (four chains of 1500 iterations each with 250 warmup iterations) and assessed convergence visually and using the R-hat statistic. All reported estimates are means of the posterior samples with the 2·5th and 97·5th percentiles of this distribution reported as the 95% CI. From the expected sensitivity of RT-PCR, we calculated the expected false-negative rate on each day (1 – sensitivity). We estimated sensitivity of RT-PCR up to a week after symptom onset because RT-PCR results after clinical diagnosis (which usually happened a few days after symptom onset) were generally not available. We also fit generalised additive models for test sensitivity as a function of the time from symptom onset (and time from last exposure to an index case) using a thin plate regression spline,^17^ as implemented in the mgcv package in R, to data form Shenzhen alone and a pooled dataset including data from Kucirka and colleagues.^5^

### Role of the funding source

WGo, who is employed by the funder of the study, contributed to the initial study concept generation, field protocol design (including sampling method planning), and laboratory test methods planning. The funder of the study had no role in data analysis, data interpretation, or writing of the report. All authors had full access to all the data in the study and had final responsibility for the decision to submit for publication.

## Results

As of Aug 1, 2020, 348 imported cases from other parts of China, 39 cases from abroad, and 75 locally transmitted cases had been detected in Shenzhen (cumulative incidence 0·35 per 10 000 population). 63 (84·0%) of 75 locally transmitted cases were close contacts of a confirmed imported case. 417 (90·3%) of the total 462 cases were confirmed during the epidemic that ended on Feb 21, 2020.

**Figure 1:**
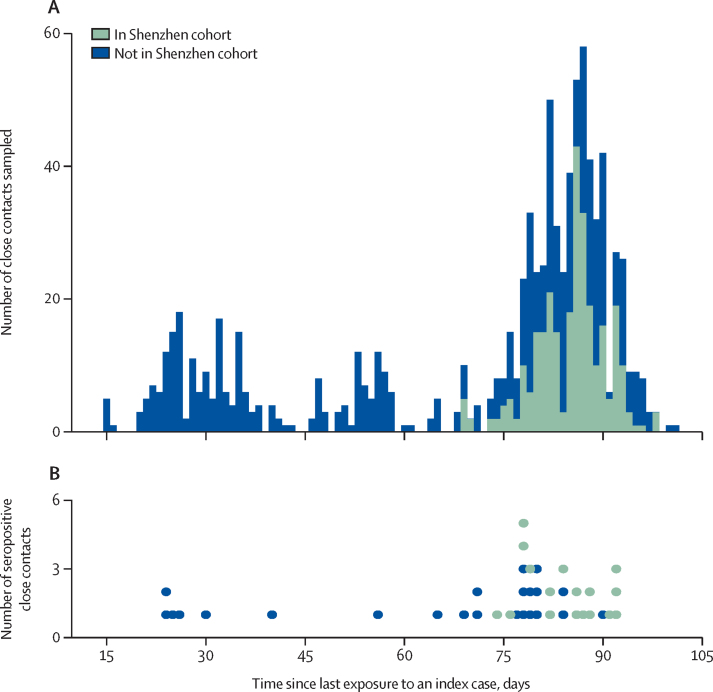
Timing of serological testing and seropositive results relative to last putative exposure Time of serological testing from last putative exposure to an index case, among all PCR-negative close contacts (A) and among those with seropositive results (B). All close contacts had one serological test each. The Shenzhen cohort was defined as individuals who were included in a previous study that characterised the epidemiology and transmission of COVID-19 in Shenzhen, by Bi and colleagues.^8^

**Figure 2:**
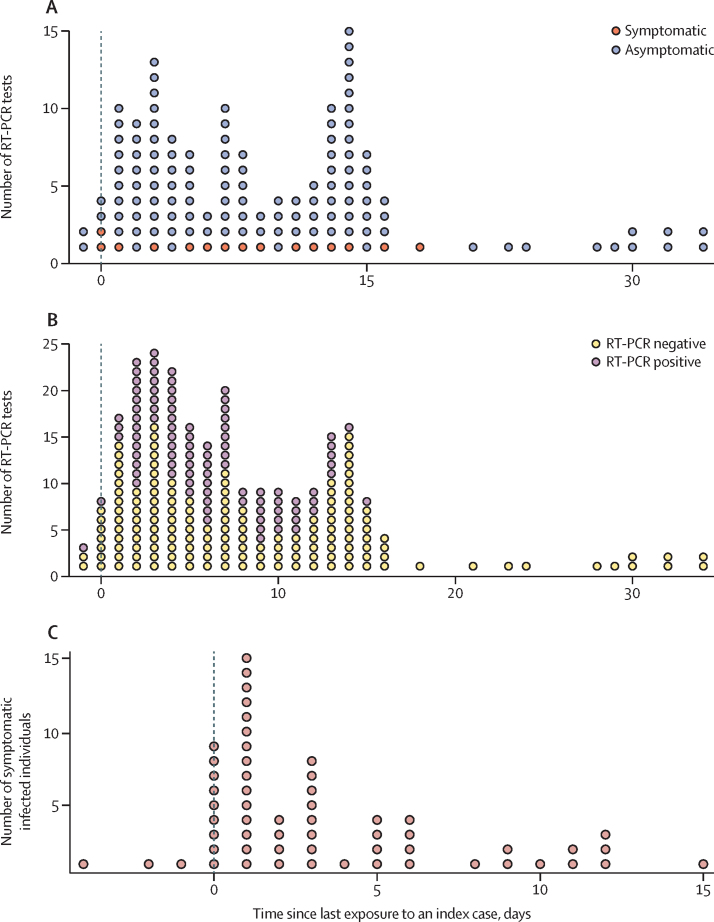
Time of RT-PCR test and time of symptom onset from last exposure to an index case (A) Time of RT-PCR tests among seropositive close contacts who were negative on RT-PCR (n=40), for those who had symptoms (n=3) and who did not have symptoms (n=37) before the end of quarantine. (B) Time of RT-PCR tests among infected close contacts who were either positive on RT-PCR (n=75) or who were negative on RT-PCR but later tested seropositive (n=40). (C) Time of symptom onset among symptomatic infected close contacts who were either positive on RT-PCR (n=55) or who were negative on RT-PCR but later tested seropositive (n=3). Two RT-PCR-positive close contacts had missing dates of last contact with an index case and were not shown in panel C. 18 individuals who were included in panel B but excluded in panel C were either asymptomatic or missing symptom onset dates.

Between April 12 and May 4, 2020, we collected serological samples from 2345 (53·0%) of 4422 RT-PCR-negative close contacts of cases of PCR-confirmed SARS-CoV-2. 1175 (50·1%) of 2345 individuals were contacts of cases diagnosed in Shenzhen that had contact tracing records. Those not included in the serosurvey were mostly non-local residents who left Shenzhen after quarantine. Sera from 880 (74·9%) of 1175 individuals were collected more than 2 weeks after last exposure to an index case (figure 1, appendix p 4). Among these 880 close contacts, the mean age was 34·1 years (IQR 24·0–44·0), and 460 (52·3%) were female (table 1, appendix p 1). RT-PCR-negative close contacts had a mean of 3·2 RT-PCR tests each (IQR 2·0–3·0; range 1·0–10·0) before the end of quarantine, and 716 (81·4%) had more than one RT-PCR test (figure 2). Most RT-PCR tests of close contacts were done at the beginning of the 2-week quarantine and near the end of the quarantine (figure 2), with the first test being done on mean average 4·5 days (IQR 2·0–7·0) after the last day of known exposure to an index case. Mean time of serological testing from last exposure to an index case was 71·4 days (IQR 56·0–87·0; figure 1). 243 (27·6%) of 880 close contacts reported frequent contact with the index case, 250 (28·4%) reported moderate contact, and 387 (44·0%) reported rare contact.

**Table 1:** Characteristics of RT-PCR-negative close contacts in Shenzhen by total antibody ELISA result

	**Seronegative (n=840)**	**Seropositive (n=40)**	**p value**
**Sex**			
Female	438 (52·1%)	22 (55·0%)	0·85
Male	402 (47·9%)	18 (45·0%)	..
**Age in years**			
0–9	75 (8·9%)	5 (12·5%)	0·73
10–19	66 (7·9%)	2 (5·0%)	..
20–29	190 (22·6%)	10 (25·0%)	..
30–39	222 (26·4%)	11 (27·5%)	..
40–49	151 (18·0%)	3 (7·5%)	..
50–59	79 (9·4%)	5 (12·5%)	..
60–69	43 (5·1%)	3 (7·5%)	..
≥70	14 (1·7%)	1 (2·5%)	..
**Symptomatic**			
No	832 (99·0%)	37 (92·5%)	0·0036
Yes	8 (1·0%)	3 (7·5%)	..
**Contact frequency**			
Rare	382 (45·5%)	5 (12·5%)	<0·0001
Moderate	247 (29·4%)	3 (7·5%)	..
Frequent	211 (25·1%)	32 (80·0%)	..
**Number of RT-PCR tests before the end of quarantine**			
Mean (IQR)	3·1 (2·0–3·0)	3·9 (2·0–5·3)	0·062
≤2	579 (68·9%)	21 (52·5%)	0·049
>2	261 (31·1%)	19 (47·5%)	..
**Days from last exposure to a case to serological testing**			
≤60	234 (27·9%)	7 (17·5%)	0·33
61–90	517 (61·5%)	29 (72·5%)	..
>90	89 (10·6%)	4 (10·0%)	..

Data are n (%), unless otherwise stated.

40 (4·5% [95% CI 3·4–6·1]) of 880 RT-PCR-negative close contacts were positive on total antibody ELISA; of these, 34 (3·9%) tested positive for IgG and 16 (1·8%) for IgM. Adjusting for assay performance,^11^, ^12^ we estimated a seropositivity rate with total antibody ELISA among RT-PCR-negative close contacts of 4·1% (95% CI 2·9–5·7; table 2), which was significantly higher than among individuals residing in neighbourhoods with no reported cases; one (0·29%) of 350 individuals was seropositive in neighbourhoods with no reported cases and none (0%) of 50 were seropositive in neighbourhoods with reported cases. The adjusted estimate for seropositivity in individuals residing in neighbourhoods with no reported cases was 0·0% (95%CI 0·0–1·1).

**Table 2:** Crude and adjusted seropositivity rates by total antibody, IgG, and IgM ELISA

	**Crude estimates**			Adjusted estimates*		
	Seropositivity rate for total antibody, n (% [95% CI])	Seropositivity rate for IgG, n (% [95% CI])	Seropositivity rate for IgM, n (% [95% CI])	Seropositivity rate for total antibody, % (95% CI)	Seropositivity rate for IgG, % (95% CI)†	Seropositivity rate for IgM, % (95% CI)
RT-PCR-negative close contacts (n=880)	40 (4·5% [3·4–6·1])	34 (3·9% [2·8–5·4])	16 (1·8% [1·1–2·9])	4·1% (2·9–5·7)	1·7% (0·0–5·3)	0·5% (0·0–1·8)
RT-PCR-negative close contacts in the Shenzhen cohort‡ (n=288)	17 (5·9% [3·7–9·2])	16 (5·6% [3·4–8·8])	9 (3·1% [1·7–5·8])	5·5% (3·2–8·9)	3·5% (0·0–8·7)	2·0% (0·4–5·1)
Individuals residing in neighbourhood without reported cases (n=350)	1 (0·3% [0·0–1·6])	0 (0·0% [0·0–1·1])	0 (0·0% [0·0–1·1])	0·0% (0·0–1·1)	0·0% (0·0–1·0)	0·0% (0·0–0·0)
Individuals residing in neighbourhood with reported cases (n=50)	0 (0·0% [0·0–1·1])	0 (0·0% [0·0–1·1])	0 (0·0% [0·0–1·1])	0·0% (0·0–6·8)	0·0% (0·0–7·0)	0·0% (0·0–6·6)

* Sensitivity and specificity of ELISA used for calculating adjusted estimates of seropositivity rates were 98% and 99% respectively for total antibody, 96% and 98% for IgG, and 90% and 99% for IgM, on the basis of results from GeurtsvanKessel and colleagues^12^ and Lassaunière and colleagues.^11^

† Sensitivity and specificity of the IgG ELISA were based on an assay validation study done by Shenzhen Centers for Disease Control and Prevention (CDC; unpublished); validation samples were collected more than 14 days after symptom onset in 23 hospitalised patients with COVID-19 and compared with 44 prepandemic controls.

‡ The Shenzhen cohort was defined as individuals who were included in a previous study that characterised the epidemiology and transmission of COVID-19 in Shenzhen, by Bi and colleagues.^8^

The unadjusted seropositivity rate was 2·9% (7 of 241 individuals) for RT-PCR-negative close contacts sampled within 60 days after the last day of exposure to a known case, 5·6% (29 of 546) for those sampled 61–90 days after the last day of exposure, and 4·3% (4 of 93) for those sampled more than 90 days after the last day of exposure (p=0·33; table 1). The overall proportion of seropositivity among RT-PCR-negative close contacts did not differ significantly by age or sex.

Only three (7·5%) of 40 seropositive RT-PCR-negative close contacts reported symptoms between the last date of putative exposure and end of the 2-week quarantine period (all three had fever, two had signs of lower respiratory tract infection, and one had nausea and headache; figure 2, table 1). Nevertheless, RT-PCR-negative close contacts who were seropositive were more likely to report symptoms than those who were seronegative (relative rate 7·9 [95% CI 1·7–27·2]; p=0·0023). In the Shenzhen cohort, RT-PCR-positive individuals were 8·0 times (95% CI 5·3–12·7; p<0·0001) more likely to report symptoms than the RT-PCR-negative individuals who seroconverted (366 [93·6%] of 391 RT-PCR-positive individuals *vs* 2 [11·8%] of 17 RT-PCR-negative seropositive individuals). Among the RT-PCR-negative close contacts in Shenzhen, including those in the Shenzhen cohort, seropositive and seronegative individuals received similar numbers of RT-PCR tests (seropositive individuals had on average 0·81 more tests [95% CI −0·04 to 1·70]; p=0·062).

The adjusted seropositivity rate for total antibody among all RT-PCR-negative close contacts was 4·1% (95% CI 2·9–5·7), which was lower than the seropositivity rate among those in the Shenzhen cohort (5·5% [95% CI 3·2–8·9]; table 2). Using the adjusted seroprevalence rate in RT-PCR-negative close contacts, we estimated that RT-PCR did not detect 36% (95% CI 28–44; 48 of 134 infections) of all infected close contacts in the Shenzhen cohort. Adjusting for these missed infections increases our estimate of the effective reproductive number by 34%, to 0·56 (95% CI 0·45–0·67). Our estimate of the secondary attack rate increased to 10·8% (95% CI 9·3–12·7) from the previous estimate of 6·6% (5·4–8·1), and our estimate of household secondary attack rate increased to 15·9% (13·4–18·8) from 11·2% (9·1–13·8).

Among the 288 individuals from the previously published Shenzhen cohort that were included in this serosurvey, we compared risk factors for seropositivity in RT-PCR-negative close contacts with the originally published results (figure 1, appendix p 2). In the Shenzhen cohort there was no significant difference in the odds ratio of testing positive for any exposure between the serological and virological studies.

**Figure 3:**
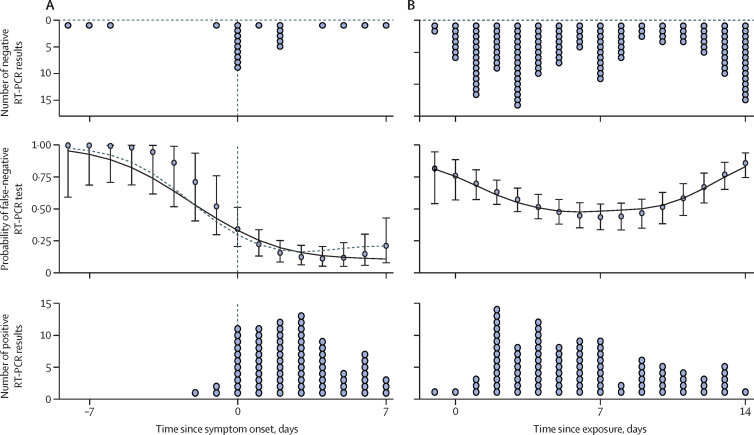
False-negative rates of RT-PCR Probability of false-negative RT-PCR test of nasopharyngeal swab, by time since symptom onset (A) and time since last exposure to an index case (B). Point estimates and 95% CIs represent estimates from the Bayesian logistic regression model for test sensitivity with a polynomial spline of third degree. The solid curve represents estimates from the generalised additive model fitted to Shenzhen data only. The dashed curve represents marginal estimates from the generalised additive model with random effects by study fitted to the combined Shenzhen data and pooled data from Kucirka and colleagues.^5^ The vertical dashed line corresponds to time of symptom onset.

We examined the variability in the false-negative rate of RT-PCR-based testing relative to the day of symptom onset using RT-PCR results from 60 symptomatic individuals who were either RT-PCR-positive (n=57) or RT-PCR-negative but later tested seropositive (n=3; figure 3A). Of the 57 RT-PCR-positive individuals, 43 (75·4%) tested positive on the first test, 12 (21·1%) on the second test, and two (3·5%) on the third test. Seven individuals had their first RT-PCR test before symptom onset and 20 had their first test on the day of symptom onset. On the basis of the best-fit polynomial spline model (three degrees of freedom; appendix p 3) we estimated the probability of a false-negative result to be 34% (95% CI 21–51) on the day of symptom onset, decreasing to a low of 11% (5–21) 4 days after symptom onset. Uncertainty in the false-negative rate is high in the days before symptom onset, but we estimate a smooth increase in the probability of false-negative results the earlier in the course of infection, reaching 100% at 5 days before symptom onset. False-negative rates were lowest at about 5–8 days after the last day of exposure to an index case, but remained above 40%, although multiple exposure events might have occurred before the last day of exposure to an index case (figure 3B).

## Discussion

The specific circumstances of the initial SARS-CoV-2 epidemic in Shenzhen, China allowed us to use serological data to gain additional insight into the epidemiology of the virus and the practical performance of RT-PCR as a surveillance tool. We found that approximately 4% of RT-PCR-negative close contacts in Shenzhen were seropositive for anti-SARS-CoV-2 antibodies, which was lower than the false-negative rate previously reported, where close contacts were tested primarily due to symptom presentation.^18^ Thus, despite a rigorous testing system, virological RT-PCR testing did not detect 30–40% of infections in Shenzhen. Even with these limitations, the serological evidence suggests that the overall control programme was successful in containing the virus; as the rate of seropositivity outside of close contacts was almost zero. Those testing negative on RT-PCR but seropositive were significantly less likely to have symptoms than those testing positive on RT-PCR, potentially reflecting difficulties in appropriately timing virological testing when there is no outward indication of the timing of viral shedding. Consistent with this theory, we found considerable variation in the chance of an infected individual testing negative on RT-PCR over the course of their infection.

It might be that RT-PCR test results are correlated with virus transmissibility, though evidence of this correlation is unclear, and we were unable to assess that in this study.^19^ Strict quarantine practices were in place in Shenzhen, requiring that individuals remained in a quarantine facility for 2 weeks from the last day of exposure, regardless of symptoms or test results. The majority (92·5%) of individuals who were seropositive but RT-PCR negative reported no symptoms. Although this observation might stem from correlations between viral shedding and development of symptoms, it could also highlight a challenge in using virological RT-PCR testing for asymptomatic surveillance. The period in which RT-PCR testing is highly sensitive is relatively short,^5^, ^6^ and sensitivity peaks around the time of, or shortly after, symptom onset. Hence, without there being symptoms as an indicator of when to test, it could be difficult to capture patients in this sensitive period. This phenomena is not unique to SARS-CoV-2, and studies of influenza and other acute respiratory viruses have shown serological attack rates of two to three times greater than virological attack rates.^20^, ^21^ Therefore, although RT-PCR is an invaluable diagnostic tool, it has important limitations as a tool for surveillance or as an outcome measure in risk-factor studies.

Our study has important limitations. We were unable to obtain serological data on RT-PCR-positive individuals, so we could not estimate the practical sensitivity of the serological tests. The sample size was relatively small, particularly for estimating risk factors in the Shenzhen cohort, and only 20% of RT-PCR-negative close contacts could be recontacted and had detailed data from the initial contact tracing. The serum samples were collected between 2 weeks and 4 months after the last day of exposure to a known case. Although we expect most individuals to seroconvert in the first month after exposure, time to sampling might not have been long enough for some close contacts to seroconvert. Although we expect cases to remain seropositive for IgG for at least 2 months after symptom onset,^10^ the post-infection dynamics among asymptomatically infected individuals is less clear.^22^ We did not adjust for longitudinal variation in ELISA performance when calculating the adjusted seropositivity rate. Although the serological tests have imperfect specificity, seroprevalence among RT-PCR-negative close contacts was much higher than the seroprevalence among those without known exposure to cases, which supports that the observed undetected infections are real. Seropositivity rate among the subset in the Shenzhen cohort is higher than the rate among all RT-PCR-negative close contacts in Shenzhen (5·5% *vs* 4·1%), possibly due to the more frequent self-reported exposure between index cases and contacts in the Shenzhen cohort. The difference in exposure possibly also explains the differences in characteristics of individuals included and not included in the cohort. The extent of undetected transmission estimated using the lower seropositivity rate in Shenzhen might underestimate the true extent.

We cannot completely rule out the possibility that seropositive individuals were exposed to an infectious individual other than their identified index case, because we did not ascertain recent travel history of these individuals. Although data on seroreversion are scarce, remaining seropositive for more than 2 months after infection is certainly plausible.^10^ However, the epicentre of COVID-19 in China was under lockdown from Jan 23 to March 25, 2020, and mandatory RT-PCR testing of all international travellers arriving in Shenzhen was enforced from March 27, 2020. As a result, the chance of these individuals travelling to high-risk areas before serological screening that was done in April was low. As the contacts tested by serology, especially those in the Shenzhen cohort, tend to be local residents, their chance of exposure outside of Shenzhen before the local outbreak started in mid-January is likely to be low. Despite the small sample size and possible selection bias that might be present in community samples without reported exposure to a known case, the low seropositivity rate in this group provides further evidence that seropositivity among close contacts was most likely due to exposure to an index case. We cannot rule out infection by an index case after the initial quarantine, as there have been reports of viral shedding after multiple negative RT-PCR tests.^23^

In conclusion, this study provides important insight into the practical limitations of RT-PCR-based virological surveillance for SARS-CoV-2. Although virological testing should remain the mainstay of disease control programmes and patient diagnostics, it, like all tools, is imperfect. Hence, it is essential that we do not rely on RT-PCR test results alone when making clinical and public health decisions. Although serological testing is an important supplement to RT-PCR for surveillance and scientific study, it is a fundamentally different tool and it cannot replace virological testing. Innovations to both improve the accuracy of virological testing and allow for more frequent, less invasive testing are, therefore, greatly needed. These data from Shenzhen highlight that even with the imperfect tools we have available, SARS-CoV-2 control is possible through rigorous surveillance, testing, isolation, and quarantine.

## Data sharing

Data and code used for generating figure 3 were available at https://github.com/HopkinsIDD/COVID19_shenzhen_sero. Other data can be made available in an aggregated format upon request to the corresponding author.
