# Supplementary material for: Insight into the practical performance of RT-PCR testing for SARS-CoV-2 using serological data: a cohort study
Source: Lancet Microbe. 2021 Feb;2(2):e79–87. doi: 10.1016/S2666-5247(20)30200-7 (PMC7816573; doi:10.1016/S2666-5247(20)30200-7)
Supplement: Supplementary appendix [file mmc1.pdf]

THE LANCET
Microbe

Supplementary appendix

This appendix formed part of the original submission and has been peer reviewed. We post it as supplied by the authors.

Supplement to: Zhang Z, Bi Q, Fang S, et al. Insight into the practical performance of RT-PCR testing for SARS-CoV-2 using serological data: a cohort study. *Lancet Microbe* 2021; published online January 19. https://doi.org/10.1016/S2666-5247(20)30200-7.

Appendix Table 1. Characteristics of PCR-negative close-contacts in the serological survey and test for heterogeneity of characteristics between PCR-negative close contact in and not in the Shenzhen cohort.

	**All PCR-negative close-contacts in the serosurvey (N=880)**	**PCR-negative close-contacts in the Shenzhen cohort (N=288)**	**PCR-negative close contacts not in the Shenzhen cohort (N=592)**	**P-value**
**Sex**				
**Female**	52·3% (460)	54·2% (156)	51·4% (304)	0·48
**Male**	47·7% (420)	45·8% (132)	48·6% (288)	-
**Age**				
**0-9 years**	9·1% (80)	12·5% (36)	7·4% (44)	0·0047
**10-19 years**	7·7% (68)	7·6% (22)	7·8% (46)	-
**20-29 years**	22·7% (200)	17·7% (51)	25·2% (149)	-
**30-39 years**	26·5% (233)	26·0% (75)	26·7% (158)	-
**40-49 years**	17·5% (154)	16·7% (48)	17·9% (106)	-
**50-59 years**	9·5% (84)	9·4% (27)	9·6% (57)	-
**60-69 years**	5·2% (46)	8·7% (25)	3·5% (21)	-
**>=70 years**	1·7% (15)	1·4% (4)	1·9% (11)	-
**Symptomatic**				
**No**	98·7% (869)	97·2% (280)	99·5% (589)	0·012
**Yes**	1·3% (11)	2·8% (8)	0·51% (3)	-
**Contact Frequency**				
**Rare**	44·0% (387)	16·3% (47)	57·4% (340)	<0·0001
**Moderate**	28·4% (250)	36·1% (104)	24·7% (146)	-
**Frequent**	27·6% (243)	47·6% (137)	17·9% (106)	-
**# of RT-PCR tests before the end of quarantine**	3·2 (IQR=2,3)	2·1 (IQR=2,2)	3·7 (IQR=2,4)	<0·0001
**<=2 tests**	68·2% (600)	87·5% (252)	58·8% (348)	<0·0001
**>2 tests**	31·8% (280)	12·5% (36)	41·2% (244)	-
**Days from last exposure to a case to serologic testing**	71·4 (IQR=56,87)	85·1 (IQR=82,88)	64·7 (IQR=36,86)	<0·0001
**<=60**	27·4% (241)	0% (0)	40·7% (241)	<0·0001
**61-90**	62·0% (546)	85·4% (246)	50·7% (300)	-
**>90**	10·6% (93)	14·5% (42)	8·6% (51)	-

Appendix Table 2. Comparison of exposure specific positivity rates in serological and virological testing, and test for heterogeneity in the exposure specific odds-ratio of positivity between serologic and virologic studies[16].

	# PCR-negative contacts with serologic testing	# Sero-positive	Seropositivity rate among PCR-negative contacts (95%CI)	Number of contacts[†]	Number of PCR-confirmed contacts[†]	P-value
**Sex**						
**Female**	156	9	5·8% (3·1, 10·5)	558	58	0·16
**Male**	132	8	6·1% (3·1, 11·5)	486	26	ref
**Age**						
**0-9 years**	36	2	5·6% (1·5, 18·1)	148	11	0·94
**10-19 years**	22	0	0	85	6	0·27
**20-29 years**	51	4	7·8% (3·1, 18·5)	114	7	0·64
**30-39 years**	75	7	9·3% (4·6, 18·0)	268	16	0·45
**40-49 years**	48	0	0	143	7	0·19
**50-59 years**	27	2	7·4% (2·1, 23·4)	110	10	ref
**60-69 years**	25	2	8·0% (2·2, 25·0)	130	20	0·64
**>=70 years**	4	0	0	72	7	0·56
**Contact Frequency**						
**Rare**	47	0	0	230	1	0·72
**Moderate**	104	2	1·9% (0·53, 6·7)	305	9	ref
**Frequent**	137	15	10·9% (6·7, 17·3)	555	71	0·76
**Contact type: household**						
**Yes**	143	13	9·1% (5·4, 14·9)	686	77	0·11
**No**	124	3	2·4% (0·83, 6·9)	456	4	ref
**Contact type: travel**						
**Yes**	54	4	7·4% (2·9, 17·6)	318	18	0·35
**No**	213	12	5·6% (3·3, 9·6)	824	63	ref
**Contact type: meal**						
**Yes**	153	12	7·8% (4·5, 13·2)	707	61	0·78
**No**	114	4	3·5% (1·4, 8·7)	435	20	ref

[†] Results from the 5th and 6th columns were reported in Table 3 of Bi *et al.*[8].

Appendix Table 3. Performance of polynomial spline models assessed with Widely Applicable Information Criterion (WAIC). Lower WAIC is consistent with better model fit.

**Degree of freedom of polynomial spline**	**Models for estimating test sensitivity from symptom onset**	**Models for estimating test sensitivity from last day of exposure to a case**	**Sensitivity analysis: models for estimating test sensitivity from symptom onset, excluding test results after the first positive test**
**3rd**	43·5	72·2	37·3
**4th**	47·9	72·8	41·6
**5th**	50·8	74·7	44·3

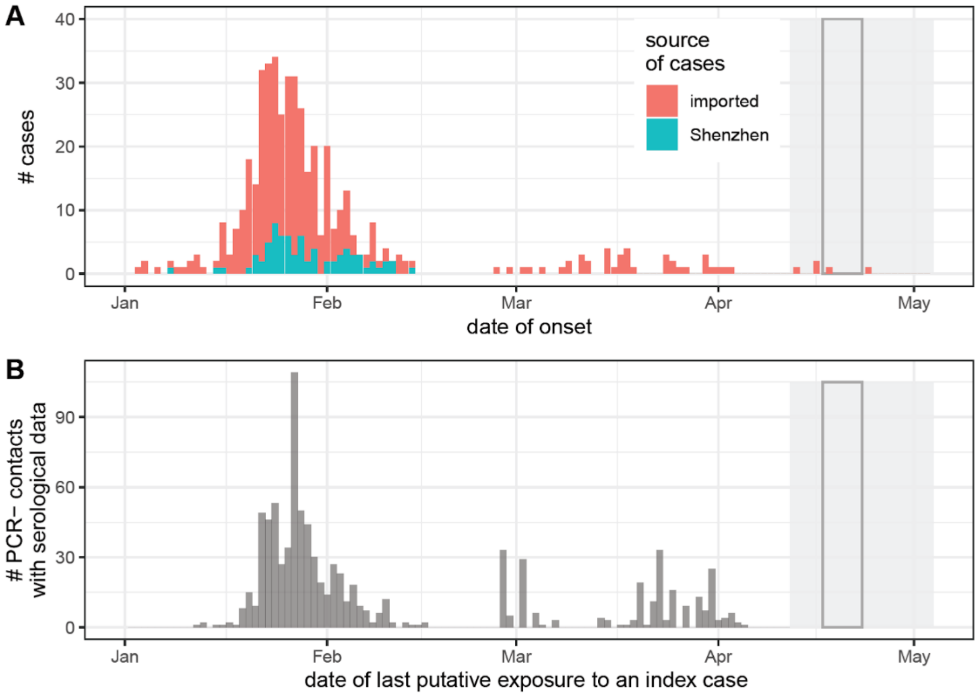

Appendix Figure 1. A) Daily imported and local cases in Shenzhen confirmed by RT-PCR. For asymptomatically infected cases, we plotted date of PCR-confirmation instead of symptom onset date. B) Date of last putative exposure of PCR-negative contacts with serological data. Shaded box represents recruitment period of the serological testing of PCR-negative close-contacts of confirmed cases. Unshaded box represents the recruitment period of the community samples without reported direct exposure

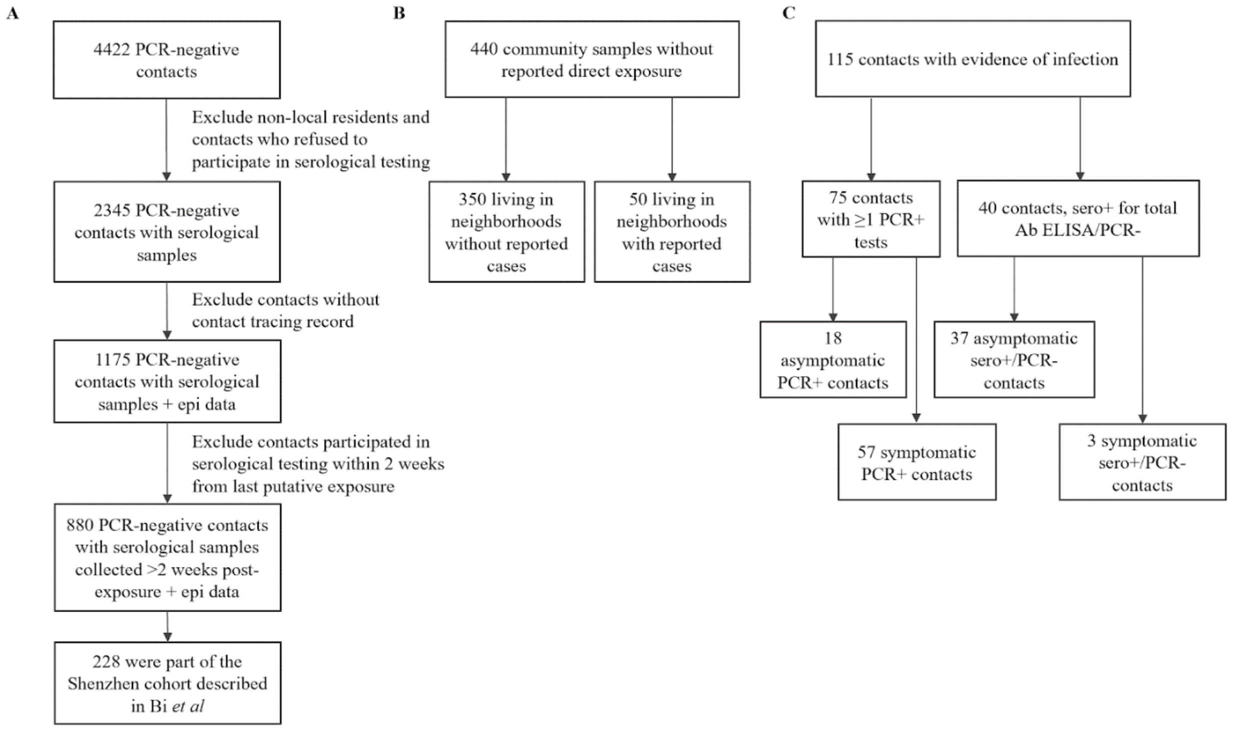

Appendix Figure 2. Sources of samples included for analyses. A) contact inclusion criteria for estimating seropositivity rate among PCR-negative contacts. B) community samples for estimating baseline community infection rate in Shenzhen. C) Contacts with evidence of infection either detected by RT-PCR during quarantine or by total Ab ELISA. RT-PCR records from these contacts with evidence of infection are used for estimating false negative rates of RT-PCR over the course of infection.

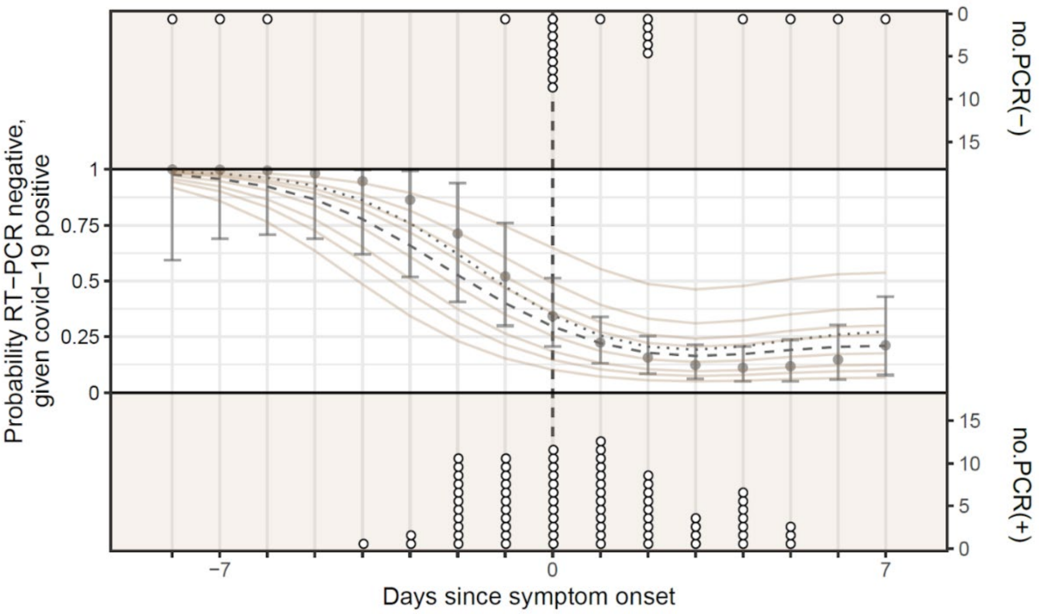

Appendix Figure 3. Heterogeneity of false negative rate of RT-PCR of nasopharyngeal swab by time since symptom onset. Point estimates and confidence intervals represent estimates from the Bayesian logistic regression model for test sensitivity with a polynomial spline of third degree. Dashed and dotted curves represent marginal estimates from the GAM model with random effect and estimates from the GAM model without random effect fitted to the combined Shenzhen data and pooled data from Kucirka *et al*.[17], respectively. Solid curves represent estimates from other studies included in Kucirka *et al*.. Dots on top and bottom of the figures indicate timing of negative and positive PCR results respectively.
